# Survival Prospects of Wild Birds Depending on the Type of Injury and Other Stressors Leading to Hospitalisation: A Long-Term (1988–2020) Retrospective Study from an Urbanised Area of the Alps

**DOI:** 10.3390/ani16020221

**Published:** 2026-01-12

**Authors:** Christiane Böhm, Molinia Wilberger, Armin Landmann

**Affiliations:** 1 Institut für Naturkunde & Ökologie, Karl-Kapferer-Straße 3, 6020 Innsbruck, Austria; chr.boehm@chello.at (C.B.); molinia.wilberger@chello.at (M.W.); 2 Innsbruck Alpenzoo, Weiherburggasse 37a, 6020 Innsbruck, Austria

**Keywords:** wildlife rescue centre, rehabilitation success, physical traumata, collisions, pet attacks, survival rates, zoo biology

## Abstract

We analysed data collected at the Innsbruck Alpenzoo (Tyrol, Austria) over 33 years (1988–2020). We examined data from 4542 wild birds of 137 species that were rescued in the increasingly urbanised and densely populated Inn Valley around Innsbruck and examined the outcome of hospital treatment (survival or death); for a subgroup of 3440 birds, we examined the length of stay at the zoo. The birds were divided into nine different groups, and the reasons for admission were divided into nine categories to analyse how the reasons for admission and membership of a bird group influences rehabilitation success and the duration of care required. Orphaned birds, birds that had become entangled in man-made structures, and birds with unknown reasons for admission had the best survival rates (60%), while birds with physical injuries, victims of collisions, and attacks by cats had the lowest survival rates (37%). Survival rates were highest among areal insectivores (66%) and waterbirds (62%), and lowest among small songbirds (45%) and woodpeckers (<39%), which suffered disproportionately from the consequences of window collisions. The overall survival rate of hospitalised birds (51%) was higher, and the duration of care required (median 11 days) was especially shorter at Innsbruck Alpenzoo than at most other rehabilitation centres. We attribute this mainly to the professional care and varied, group-specific diet provided to the patients, which we describe in detail. We also discuss the problems and limitations of wild bird care for zoo staff in addition to their daily tasks. Thereby, it became apparent that the retirement of experienced bird carers at the beginning of the study period and the subsequent steady changeover of staff members had a negative impact on success rates.

## Simple Summary

We analysed the Innsbruck Alpenzoo database of wild birds that were rescued in the densely populated Inn Valley around Innsbruck, Austria, and admitted to the zoo over a period of 33 years (1988–2020). In this publication, we focus on the outcomes of rehabilitation efforts by the zoo staff. Our objectives are (a) to explore the general survival chances of different bird groups, and (b) to examine how the reasons for admission influence rehabilitation success in terms of survival rates and the duration of care required. Orphaned birds, specimens that had become entangled in man-made structures, and birds with unknown reasons for admission had the best survival rates, while birds with severe physical injuries, victims of window collisions, and cat attacks had the lowest survival rates. Rates were highest among areal insectivores and waterbirds, and lowest among small songbirds and woodpeckers, which suffered disproportionately from the consequences of collisions. The overall survival rate of hospitalised birds was higher, and the duration of care required was shorter at Innsbruck Alpenzoo than at most other rehabilitation centres. We attribute this mainly to the professional care and varied diet provided to the patients. We also discuss the problems and limitations of wild bird care for zoo staff in addition to their daily tasks.

## 1. Introduction

Wildlife rescue and rehabilitation centres (WRCs) have been operating worldwide for decades. This is particularly true in many industrialised countries in North America (e.g., [1,2,3]), Europe (e.g., [4,5,6,7,8,9]) and Australasia (e.g., [10,11,12,13]), but also in Latin America (e.g., [14,15]) and other regions (for overviews, see [3,16,17]). These WRCs admit large numbers of wild birds, resulting in potentially millions of individuals being cared for and released each year worldwide [16,17]. Data from WRCs can provide important insights into the immediate causes of morbidity and mortality in birds in human-dominated landscapes, as well as in the outcomes of animal welfare and veterinary interventions.

Despite growing recognition of the value of WRC data within the scientific community [5,9,17], this data remains a largely underutilised source of information, and there is still much untapped potential for research based on wildlife rescues. A major reason for this gap may be that WRCs are often run by volunteers or animal caretakers who have limited time, money, or scientific expertise to invest in standardised collection and analysis of the data available to them [8]. Well-founded published studies based on information from rescue databases are therefore still relatively rare and/or tend to be limited to certain taxa, with a clear focus on charismatic larger and/or endangered bird taxa such as birds of prey (in Europe, e.g., [3,5,18,19,20,21,22]) or seabirds ([23]; for overview, see [3]), or on characterising the causes and species-level demographics of admissions ([1,8,17] with further references).

Retrospective data sets from European and North American WRCs consistently identify trauma (vehicle collisions, collisions with buildings/windows, cat attacks, electrocution, gunshot wounds) and orphanage as the most common causes for bird admissions, albeit with significant regional, species-specific, and seasonal differences. However, long-term studies (i.e., covering a period of more than ten years) that include the entire regional bird fauna and examine the causes of hazards to birds and the outcomes of rehabilitation remain very rare, especially in Europe [4,6,8,9,24], and are almost completely lacking for densely populated Central European study areas [8,9,24].

Here, we present a retrospective analysis of long-term data (1988–2020) from a rescue database at the Innsbruck Alpenzoo, Austria, which (until the COVID restrictions) also functioned as a WRC [8,24,25]. The data were collected by trained bird care staff and monitored and organised by professional ornithologists and zoo veterinarians. In previous work, we focused on faunistic aspects [25] and, more recently, on the causes and demographics of admissions at the species level [8]. In this study, we analyse the outcomes of rehabilitation efforts mainly in terms of survival rates and the probability of release. Specifically, we examine the interaction between the reason for admission, the type of injury, and stress factors before and during hospitalisation, the duration of rehabilitation, and the species-specific characteristics of the birds (age, size, food preferences, taxonomic affiliation).

Given the growing urbanisation and human-induced threats, we believe it is increasingly important to clarify these relationships in order to ensure the welfare of patients and the protection of birds and to draw conclusions for future rehabilitation strategies, e.g., to develop evidence-based guidance for triage and resource allocation, which is an issue that is becoming mountingly important for WRCs [3,6,9,16,17].

## 2. Materials and Methods

### 2.1. Study Area and Origin of Rescued Birds

The federal Austrian state of Tyrol (12,648 km^2^) lies at the heart of the Alps and is dominated by mountainous terrain. The Tyrolean capital, Innsbruck, with around 132,000 (2024) inhabitants (city centre at 575 m above sea level, built-up area between 565 and 740 m asl) lies in the centre of the densely populated Inn Valley, and the Innsbruck Alpenzoo is located on a southern slope on the northern outskirts of the city (details see [8]). Most hospitalised birds came from an area nearby (distances < 25 km from the Alpenzoo). Most birds (83%) were rescued in the city of Innsbruck and adjoining suburban areas itself, and another 13% came from the near central Tyrolean Inn Valley and nearby terrasses [8,25]. Our data therefore mainly reflect problems faced by birds in urbanised landscapes heavily influenced by human structures and activities.

### 2.2. Bird Admittance Procedure

Being one of the few zoos where regional visitors can drop off wild orphaned, weak, or injured birds, the Innsbruck Alpenzoo has seized this opportunity to collect data about such birds via a standardised admittance procedure since 1988 up till 2020. Unfortunately, due to COVID restrictions and bird flu problems, only small numbers of birds were hospitalised in subsequent years. The limited data available from 2021 onwards is therefore not included here. The admission procedure, admission causes, and demography were already described in detail in [8]. Besides of the circumstances of discovery, the age, and sex of birds, the data sets include the general condition (health state) and the type and severity of injuries sustained by the birds. Infection in our data set could not be defined as a separate admittance category (but see, e.g., [9]) because only a small proportion of the birds admitted were specifically examined in a laboratory.

### 2.3. Hospitalisation, Care, and Release Procedures

When a rescued bird was taken in, its species, age (nestling/fledgling vs. adult), general condition, and visible trauma (if any) were recorded, and bird keepers, bird curators, and veterinarians jointly decided on the further course of action, care, and/or treatment.

Until 2012, the Alpenzoo only had a vet on call, and it was not until 2013 that a vet was available on a daily basis. Therefore (as already stated above), blood samples were only rarely taken to test for parasites and/or diseases, and in most cases, there are no specific veterinary records available. Accordingly, the staff at the Alpenzoo did not carry out any special medical treatments and the general treatment of rescued birds consisted mainly of good care, accommodation, and appropriate feeding. However, if a fracture was suspected, the bird was X-rayed. If necessary, fracture surgery was performed or a splint was applied, and only in recent years (since 2013) have painkillers been administered for serious injuries. In rare cases of severe injury or illness with no realistic chance of successful rehabilitation, the birds were euthanised at the day of admittance, and these birds are not included in the analyses.

All rescued birds were always separated from the exhibition area and contact with the “zoo” birds were avoided for hygienic reasons. Nestlings and newly fledged birds were taken into a special rearing station, placed into artificial nests or smaller aviaries, and reared with special food and fed at least every 40 min by the staff. Adult rescued birds were brought into aviaries of a specialised quarantine station. They stayed there until they died or could be released. Most of them stayed for several days or, in some rare cases, weeks, depending on their condition or severity of injury. Birds with leg, wing, body or head injuries (fractures), or other unclear traumata were first treated by the zoo veterinarian then cared for by bird keepers in the quarantine station.

The small care aviaries were equipped with natural structures such as branches for perching and hiding, and shallow bowls with water, soil, or sand. The enclosures were well-lit, with only low lighting at night for orientation. The size of the enclosures was always selected with consideration for the respective species and the best possible handling. Birds with injuries (fractures) to their legs, wings, body, or head, or with other unclear traumas, were initially deliberately kept in small enclosures (i.e., small passerines in 1 × 1 × 1 m) or even in cardboard boxes with nets to prevent further injury. Birds that stayed longer than five days and/or recovered quickly and behaved normally were moved to larger enclosures (3 × 3 × 2 m) that were equipped according to the requirements of their species. The same applied to the nestlings.

Emaciated, exhausted (“weak”) birds with no evident injuries or causes for weakness were given basic care (water, appropriate, easy digestible food) and initially kept in smaller aviaries, where they could hide, calm down, and recover, and could be more easily monitored. If their general condition quickly and clearly improved, these animals were released on the same day or the following days, if possible, or otherwise kept in care until they fully recovered. Carnivorous birds (raptors, herons, grebes, etc.) were supplied with mice and rats from the zoo’s own breeding station, with one-day-old chicken or with fish accordingly. The zoo stuff did not provide any commercially available food, as wild birds would hardly accept it anyway. Living food which moves is a good incentive for injured birds to accept food. Insectivorous small birds therefore were given a varied diet from the zoo’s specialised insect breeding programme (crickets, wax moth, larvae of beetles, meal worms *Tenebrio molitor*, king worms *Zophobas morio*) and minced beef heart mixed with self-cooked curd cheese as well as ant pupae. Nestlings received pulverised snail shells as a source of calcium. Herbivorous species and seed eaters were given high-quality standard seed mixtures available from specialised stores as well as fresh seedlings collected in the zoo area, e.g., dandelions (*Taraxacum officinále* agg.), which are particularly important for rearing young birds. For the rearing of finches, specialised food additions were used. Fresh fruits, carrots, insects, meat, and mice were fed to the omnivorous species.

As soon as adult birds and fledglings were able to feed themselves and fly, became independent, behaved normal, and reached stabilised species-specific body mass, they were released at forest edges or other appropriate habitats in the surrounding of the zoo or at nearby water bodies (e.g., river “Inn”, ponds). Some healthy birds were also given to partner zoos and certified bird keepers in compliance with regional laws and CITES regulations. These birds were also considered “released”. Only since 2020 have released birds been ringed in cooperation with the Austrian Bird Observatory to provide information on the survival chances of birds that have been cared for and released into the cultural landscape (compare [9] with a similar approach). However, there have been too few recoveries to date to be able to evaluate these data here.

### 2.4. Data Stock, Data Preparation

Over a period of 33 years (1988–2020), the Alpenzoo has admitted 5301 wild bird individuals from the study area. These birds belong to 145 species (cf. [8,24,25]). The original data were checked for accuracy and consistent formatting, and records with large gaps, unclear and uncertain entries, and data belonging to captive refugee bird individuals, such as feral pigeons (*Columba livia var. domestica*), or species brought in from outside our study area were omitted. After hospitalisation, records were subsequently also kept of failures and successes of care measures (death or recovery) and of the duration until release or death. Caring for the birds brought in by the public is a time-consuming additional task in the everyday work of the zoo staff. Unfortunately, this means that individual data sets are often incomplete and there are regrettable gaps in the documentation on the fate of hospitalised specimens (either deceased or released) and, in particular, on the duration (number of days) that individual birds were in care. As a result, the sample size that can be analysed varies for individual aspects or species.

The zoo database was supplemented by us for this study with species-specific classifications. We assigned each species to one of six body size classes (using mean adult body weights from the literature); for definitions and class boundaries, see [8]. Furthermore, to investigate whether the systematic position of a bird and/or ecological characteristics influence the probability of survival under care and duration of hospitalisation, we assigned most (93% of 4542 individuals) bird species to one of six different systematic groups and/or ecological guilds. A further 7% (315 individuals) belonged to 17 different families (12 bird orders) of Nonpasserines and to various ecological guilds. We therefore roughly divided this heterogeneous group into three weight classes based on adult weight (<250 g, 250–1000 g, >1000 g) and compared their survival rates with those of more specifically defined groups. The bird grouping does not strictly follow systematic criteria. It also considers the available sample sizes as well as practical requirements for care and accommodation, which are determined, among other things, by body size, diet, or even group-specific types of injury. For example, swallows are not included in the group “Small Passerines” but are grouped together with swifts in a guild of areal insectivores which share similar reasons for admission and care requirements. Similarly, woodpeckers, which mainly are brought in as victims of window collisions, are treated as separate group and not included in the group “Small Nonpasserines”. In addition, Accipitriformes, Falconiformes, and Strigiformes are grouped together into a “raptor” guild.

To examine the influence of the severity and type of the most significant distinguishable physical injuries on the probability of survival, we separately analysed physical trauma according to affected body regions and organ systems, based on the veterinary findings. We therefore distinguish between injuries of the respiratory system, leg, body, head injuries, and feather damages, and compare the proportion of successful treatments of such traumata with the survival prospects of birds with other admissions causes.

Rearing of nestlings and freshly fledged juveniles of herbivorous or seed-eating species, which initially require insects as food but later increasingly need soft seeds at least from the 10th to 12th day onwards, is generally considered to be more difficult than rearing mainly insectivorous species (Ellen Thaler pers. com.). We therefore analysed prospects of small songbirds (including swifts) to see whether there were differences in care success between young and adult birds of these two groups.

### 2.5. Data Analysis and Statistical Treatment

To assess differences in survival rates (i.e., the probability of a bird being released) between the various causes of admission and between the bird groups distinguished, we first performed pairwise chi^2^-tests with Yates’s correction using the numbers of surviving vs. deceased birds in each category of admission cause/bird group as variables. The same was performed to test differences between types of physical traumata. In addition, we grouped the basic outcomes of hospitalisation for each individual bird into two categories by creating a binary variable “survival”” with 1 = bird deceased and 2 = bird released (see similar approaches in [5,9]). We choose 1 instead of 0 for deceased birds to avoid possible divisions by zero during testing. To reduce the influence of individual dominant species on the results, we used the mean values for each species which varied from 1.0 to 2.0 within compared species groups as the basis for statistical testing. The results thus can be directly transformed to “survival rates” in figures (e.g., 1 = 0%, 1.5 = 50%, 2 = 100% survival rate).

The mean and standard deviations calculated for each reason for admission and for each species in each bird group were then tested for significant differences using standard univariate test statistics (One Way Analysis of Variance, Pairwise Multiple Comparison Procedures (Holm–Sidak method), Mann–Whitney Rank Sum tests, and Kruskal–Wallis One Way Analysis of Variance on Ranks were used when normality tests (Shapiro–Wilk) failed, e.g., to investigate differences in the mean duration of care (number of days) until release among the treatment groups). Thereby, duration was set at 0.5 days for birds which could be released after a few hours on the same day of admission after receiving basic care (see above). To avoid spurious correlations when multiple pairwise testing was performed, we used adjusted *p*-values by applying the FDR method which not only reduces false positives but also minimises false negatives [26].

Differences in the duration of care were not tested between groups for birds that deceased during hospitalisation because the medians and median absolute deviation values did not differ between the groups. However, we report descriptive metrics for the care duration until death for this group.

Like a recent study with similar data [9], which is particularly interesting for comparison purposes as it comes from another urbanised area in Central Europe, we also explored and discuss whether the survival probability of hospitalised birds and the duration of their rehabilitation are influenced by the interaction between the cause of admission and the birds’ affiliation to systematic groups and/or ecological guilds. However, we refrain from GLM-modelling survival probabilities as a response variable using the interaction between the causes for admission and the affiliation of bird groups (see [6,9]), as we believe that the interpretation of such multivariate models is quite complicated and the results are not immediately transparent and of limited use for bird care practice.

Data were arranged and pre-analysed with Excel (version 16 for Windows Office 365, Microsoft, Redmont, WA, USA). For graphics and statistical analyses, the tools in SigmaPlot 12.0. (Systat, Chicago, IL, USA) were used.

## 3. Results

### 3.1. Survival Rates of Hospitalised Birds Depending on Causes of Admission

Overall, data on the basic outcome of hospital treatment is missing for approximately 14.3% of the 5301 birds admitted to the Alpenzoo between 1988 and 2020 (Table 1). Data on the duration of rehabilitation are not available for 29.4% of the 2313 birds released and are missing on the duration of care for 18.9% of the 2229 birds that did not survive.

For 4542 or 85.6% of all bird individuals admitted to the Alpenzoo from 1988 to 2020 (see [8]) and which overall belong to 137 wild bird species, at least the basic outcome of rehabilitation (deceased/released) is known. For more than a quarter of these birds (28%) which were taken into care without diagnosed injuries or traumata, no particular cause for admittance was stated or registered in the protocol and these cases thus are termed “undetermined” here. Orphaned nestlings (31.8%), victims of window strikes (12.7%), and (to a lesser degree) victims of cat, dog, or raptor attacks (6.2%) were the most common specific individual causes for admissions. Two other common but less specific causes for hospitalisation were general exhaustion, termed “weakness” (6.5%), i.e., mostly emaciated and/or apathic birds with signs of dehydration, starvation, but unclear primary causes, and various physical traumata (12.3%). Other causes of admission are less important with a share of less than 2.5% of all cases (for details, compare with Table 1).

**Figure 1:**
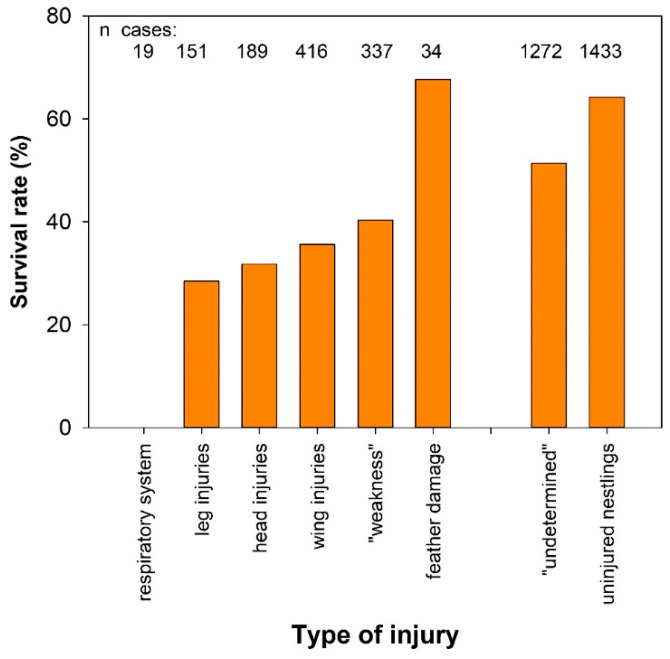
Survival rates of birds admitted to the Innsbruck Alpenzoo with different physical injuries and different affected body regions compared to some other main causes of admission without obvious injuries. *n* cases = sample sizes available for comparisons (see Table S2 for significance levels of differences).

Without taking into account that, for most causes for admission, only a few individual species are responsible for the majority of cases, there are significant differences between the causes in terms of the success of the care provided and the probability of survival (Table 1 and Table S1). Of all admissions with documented rehabilitation outcomes, a total of 51% resulted in the survival and release of the birds, while 49% died or (in rare individual cases) had to be euthanised (Table 1). However, of all the main reasons for admission, the hospitalisation and care of orphaned nestlings (or dependent young birds) achieved the highest success rate with an overall survival rate of 63.4%, which is significantly higher than all other main admission reasons, with the exception of ‘undetermined’ reasons, which in turn also differed significantly from other main causes with larger sample sizes (see Figure 1 and Table S1). In contrast, the chances of survival for injured or traumatised birds after attacks by domestic animals (mainly cats) or raptors (birds of prey, owls, corvids) were the lowest, followed by birds admitted with physical trauma from various other causes (35.7%), and victims of vehicle collisions. The latter did not differ significantly from victims of collisions with buildings (mostly window strikes; for survival rate; see Table 1; for significance levels, see Table S1).

Within the 33 years analysed, the total number of birds admitted, and most causes of admission increased (Table 2; see [8]). As conditions for bird care may have changed over such a long period, we also investigated whether the survival rates of birds admitted for different main causes varied between the periods (Table 2). While there were no significant changes between the last two decades, hospital stays for birds in the early phase of the project (1988–1999) had higher overall success rates. This is particularly evident in the care of “weak” birds and, to a lesser extent, in nestlings and victims of building collisions (window strikes), which is a reason for admission that has increased significantly, especially in the last decade analysed (2011–2020) (Table 2).

### 3.2. Influence of the Type of Injury on the Survival Rate of Hospitalised Birds

The probability of survival for birds also depends on the severity and type of physical injuries as well as the affected body regions and organ systems (Figure 1). We investigated whether there are significant differences in survival rates between injuries to the respiratory system, leg, body and head fractures, and feather damages, and compared these traumas with the survival rates of other main causes of admission for birds without obvious physical injuries. Although we have little data on cases of injuries to the air sac system or lungs, the chances of survival for such birds appear to be extremely low; all 19 individuals (mostly small songbirds but also including two Kestrels *Falco tinnunculus* and one Golden Eagle *Aquila chrysaetos*) died within one or two days (and in one case, six days) despite attempts to care for them after admission. Survival rates of leg-, head-, and wing injuries did not differ significantly from one another but were significantly lower than those of birds with feather damages, with “undetermined” problems and compared to nestlings without physical trauma (*p* < 0.001; chi^2^ tests). In addition, the proportion of surviving birds were also lower for birds with leg injuries compared to “weak” birds (*p* < 0.05), and for “weak” birds compared to birds with feather damages (*p* < 0.05; see also Table S2 for differences between “weak”, “undetermined,” and nestling cases).

### 3.3. Differences in Rehabilitation/Care Duration

Across all causes of admission and including all species, birds that did not survive the rehabilitation process had a mean care duration of only 1 ± 0.5 days, as the length of care for birds that died on the day of admission (despite care) was included in the calculation with a value of 0.5 and accounted for more than 50% of cases in all groups. Furthermore, although our records show longer care periods for birds that ultimately died (maximum 125 days), even documented care periods of more than 10 days account for less than 10% (174 out of 1807 cases), and care periods of more than one month are very rare in our sample (2%, 37 cases). Meaningful statistical comparisons between the reasons for admission or bird groups are therefore not possible for deceased birds (Table 1).

However, there are differences in the length of rehabilitation between the main causes for the admission of surviving birds. Orphaned or stranded nestlings, birds with physical injuries, and victims of attacks by cats, dogs, or wild raptors required the longest care and had to be looked after for an average of about two weeks before they could be released, which is significantly longer than for most other reasons for admission (see Table 1, cf. Table S2 for significance levels). Although the average length of rehabilitation was highest for orphaned birds (median 15.5 days), the proportion of birds that required longer-term care was higher for other admission causes. Rehabilitation took longer than one month in 21% of all documented 156 cases of birds with physical injuries, and longer than two months in 10% of all such cases. In contrast, only 12% of 644 nestlings were in care for longer than one month and only 1% longer than two months. Significantly shorter rehabilitation times than for the three reasons mentioned above were documented especially for victims of window collisions, with only three days on average between admission and release (see Table 1 and Table S2). The proportion of longer care periods has also been low (9% of 193 cases longer than one month, only two cases longer than two months) for birds with this admission cause.

### 3.4. Differences in Survival Rates Among Various Bird Groups in Care

**Figure 2:**
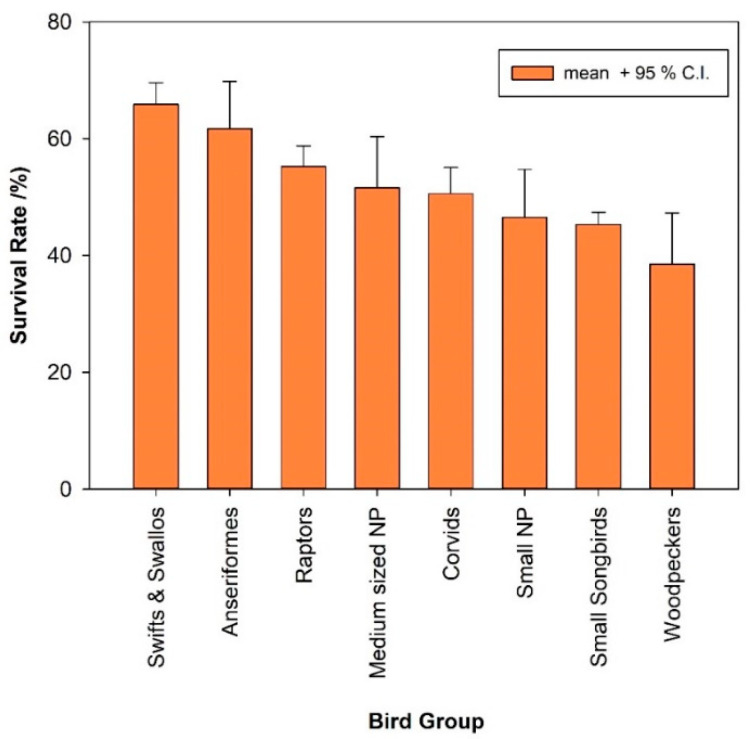
Chances of survival of birds belonging to different systematic/ecological groups cared for at Innsbruck Alpenzoo. Only groups with more than 100 cases are depicted. Number of bird species involved in each group, sample sizes and data for larger nonpasserine birds; see Table 3. Significance levels of differences between groups cf. Table S3.

The care for hospitalised small areal insectivores yielded the highest rehabilitation success with about two thirds of admitted birds surviving (Figure 2, Table 3). The overall survival rate of hospitalisation in swifts and swallows is significantly higher than that of all other bird groups, except of Anseriformes (62% survival rate). Raptors and Corvids are other bird groups where more than 50% of hospitalised specimens survived (see Table 3; for significance levels of differences to other bird groups, compare with Table S3). In contrast, the chances of survival for injured or traumatised small species either of Nonpasserines (including Woodpeckers, separated here as an own group) or small songbirds (except swallows) are on average lower than that of all other groups (Figure 2, Table 3). Woodpeckers (with the Great Spotted Woodpecker *Dendrocopus major* dominating the sample with 71% of all woodpecker admissions, Table 3), in particular, had low survival prospects (38.5%) which differed significantly from those of most other groups (Table S3).

### 3.5. Survival Prospects of Insectivorous vs. Herbivorous Small Birds

The nutritional needs of species may influence the success of care even in a zoo with professional bird carers, and with the resources to provide varied and species-appropriate food for its animals. Because sample sizes were appropriate, we used a subset of small songbirds (including Common Swifts), which are a comparatively homogenous group regarding body weights (<100 g), to investigate whether there are differences in the rehabilitation success between species that (at least as adults) prefer mainly plant-based food and those that feed mainly on insects (including spiders and earth-worms). In addition, the survival rates of young birds and adult birds from these two groups were compared. Only data from species with at least five birds in care were used, and the sample comprises six species of Finches (*Carduelis chloris*, *C. carduelis*, *Coccothraustes coccothraustes*, *Fringilla coelebs*, *Pyrrhula pyrrhula*, *Spinus spinus*), four Tits (*Cyanistes caeruleus*, *Lophophanes cristatus*, *Parus major*, *Periparus ater*), two Sparrows (*Passer domesticus*, *P. montanus*), and Nuthatch (*Sitta europaea*); as for insectivorous species, it included three species of Warblers (*Sylvia atricapilla*, *Phylloscopus collybita*, *Ph. trochilus*), four Thrushes (*Turdus merula*, *T. philomelos*, *T. pilaris*, *T. viscivorus*), European Robin *Erithacus rubecula*, Common Redstart *Phoenicurus phoenicurus*, Black Redstart *Ph. ochruros*, European Pied Flycatcher *Ficedula hypoleuca*, European Wren *Troglodytes troglodytes*, Pied Wagtail *Motacilla alba*, and Common Swift *Apus apus*.

**Figure 3:**
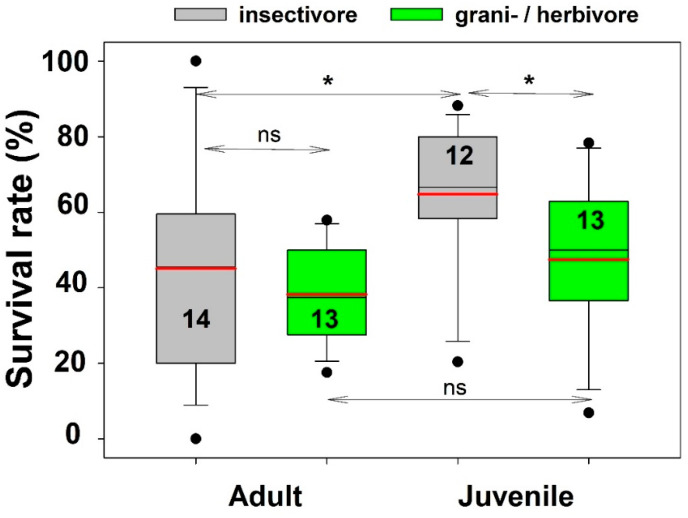
Differences in survival rates of adult and juvenile small birds with different adult diets that were hospitalised at Innsbruck Alpenzoo. Number of different species (= numbers inside the boxes). Sample sizes (number of individuals): adult/juvenile insectivores; 434/1417; adult/juvenile herbivores: 199/825. Mean and median values (black/red lines); 25–75% percentiles (boxes); 10, 90% percentiles (whiskers) and outliers (dots) are shown. Significant differences = * (*p* < 0.05; two-tailed *t*-tests).

Overall, insectivorous species had higher survival rates than herbivorous birds on average, but these differences were only significant between juveniles of both groups. In addition, survival prospects of insectivorous birds in care were significantly lower for adults compared to juveniles (Figure 3).

## 4. Discussion

In this study, we analyse the results of rehabilitation efforts by the staff at Innsbruck Alpenzoo for 4542 injured, orphaned, disoriented, or weakened wild birds from 137 species that were rescued by members of the public and brought to the zoo between 1980 and 2020. We define “success” here from a wildlife rehabilitator’s perspective [17], i.e., when a bird has recovered from the initial problems or injuries that led to its admission to such an extent that it can be returned to the wild. Failure, on the other hand, means that a bird died during care or had to be euthanised because there was no prospect of recovery. We therefore simply calculate the ‘survival rates’ as the proportion of birds that could be released after care compared to the number of individuals that died.

### 4.1. Methodological Aspects

According to [16], studies analysing causes and outcomes of rehabilitation efforts averaged 8.9 years in duration and included on average only 26.9 species including not only birds, but also other vertebrates (e.g., [6,11,15]). The maximum duration of studies dealing with rehabilitation outcomes, including urbanised areas in Europe, is 19 and 20 years [5,9,27], and to our knowledge, only three real long-term studies exist, including songbirds and a wide array of other bird groups [6,9,27]. Our study, on the other hand, not only analyses data spanning more than three decades, but also covers the entire spectrum of bird species occurring in the region [8,24,25]. This heterogeneity and diversity of the material pose some problems in interpreting the results. As we have documented in [8] (see also Table 2) both, the total number of birds as well as the number of bird species admitted have increased since the start of the data sampling, whereby orphaned nestlings and victims of glass collisions were the most common specific reasons for admission and mainly responsible for the increase (see similar trends in [5,6,7,26,27,28]). In addition, the annual number of birds hospitalised with distinctive (and in many cases, severe) physical traumata has tripled from the first period (1988–1999; 9.5 cases/year) to the last decade (2011–2020; 28.9 cases/year—see Table 2), even when injuries from collisions and cat attacks are not included.

As already analysed in [8], we think that this increase is mainly due to different external causes: Firstly, the landscape of the Inn valley around Innsbruck has undergone a rapid (sub)urbanisation in the last few decades, adjoined with a strong increase in built-up areas and buildings in the vicinity of Innsbruck, including a rapid transformation of farming villages into suburban-like residential areas in the vicinity of the city [29,30]. Secondly, we think that emotional attitudes of people towards nature and animals have changed as well. Thus, the proportion of people keeping cats and other pets as well as the motivation of people to help injured wild animals and to bring dependent young birds and nestlings to a WRC has increased [31]. In addition, personnel, space, and budget resources and workloads at a zoo will also change over a long period of time, thereby influencing the success of care and the survival rates of hospitalised animals. In the case of Innsbruck Alpenzoo, the number and size of exhibition aviaries have increased in recent decades, but the number of bird keepers has not risen accordingly, despite the increasing workload and the growing number of wild birds they care for. Even more challenging was the fact that long-serving, experienced staff retired and were replaced by younger bird keepers or seasonal helpers who also changed more frequently than at the beginning of the study period, resulting in little continuity in care procedures (personal experience of C.B. as bird curator in charge).

We have demonstrated that there has been a decrease in the care success (survival rates) for admitted birds from 1988 to 1999 to 2010 to 2020 (Table 2). In our opinion, it is no coincidence that particularly time-consuming hospitalisation cases (nestlings, weakened birds) have recently had significantly lower success rates than in the past. Although admission rates for species with special care needs have also changed slightly in recent decades and probably interact in complex ways with these zoo-specific changes, we generally assume that the latter are more important in explaining the patterns shown in Table 2. However, we generally emphasise that it is difficult to compare the survival rates documented here with those from other studies (e.g., [2,7,9,27,28]) and that the respective results should be interpreted with caution. This is because survival rates during rehabilitation vary not only depending on the bird group, the specific characteristics of the species being cared for (e.g., body size, nutritional requirements), and the location of the study, but also because the various WRCs differ in their admission and release protocols, animal monitoring, and the experience and resources available [6,17,32].

### 4.2. Outcomes of Rehabilitation Depending on Causes of Admission

Between 1988 and 2020, slightly more than half (51%) of the birds hospitalised at Innsbruck Alpenzoo could be released, regardless of the reason for their admission. Survival rates for the various reasons for admission ranged from 63.4% (orphaned/stranded birds) to 27.1% (victims of attacks by cats, dogs, and raptors). The rates were above average (55.3%) for birds with ‘undetermined’ reasons for admission (28% of all documented cases), which in our opinion suggests that this group also includes many young birds and animals with less obvious injuries (see also [9] with similar arguments).

As discussed above, comparing release rates from WRCs is problematic. The problem is exacerbated by the fact that most studies that refer to larger data sets also include other vertebrates, whose survival rates generally differ from those of birds [1,6,7,27,28,33], and do not always clearly distinguish between the release rates of the different animal groups. The overall survival rates at the Alpenzoo appear to be higher than the average rate of around 40% reported by ‘classic’ WRCs {6, 27], but the release rates of birds reported in other recent studies in Europe with a higher proportion of songbirds in the sample vary between 40% [7], 54% [33], and 71% [9]. Regardless of the overall differences, all of the above studies cited above report large differences in the survival/mortality rate of the birds taken in, depending on the reason for their admission, with the following trends consistently apparent: The care of orphaned birds (nestlings or fledglings) achieves the best results, with a release rate of usually close to or above 60% and up to 79% [9] or even 84% ([5] for raptor species). Even in WRC studies that report very low overall release rates, the care of orphaned birds was much more successful than for other reasons for admission (e.g., [22] for various raptors, [34] for Kestrels or [35] for Wood Pigeons). On the other hand, mortality rates of well below 50% appear to be common among birds that are brought in after collisions with buildings (e.g., [2]) or vehicles. They are particularly high among victims of attacks by domestic animals or wild raptors, with cats being responsible for the vast majority of these cases, at least in urban areas. It is noteworthy that not only small songbirds and small Nonpasserines suffer from cat attacks, but also stronger and larger species such as corvids, owls, and birds of prey.

The fact that, regardless of the size of the birds, often only about one-third or even only about one-quarter of victims of cat attacks survive hospitalisation (this study [1,5,6,33,34]) shows that even individuals with minor physical injuries have problems surviving due to infections. Only immediate treatment with antibiotics can improve the chances of survival for such birds (personal experience C.B., personal comm. of zoo veterinarians).

Many studies simply refer to ‘trauma’ or unspecified ‘other injuries’ sustained by rescued birds as the reason for hospitalisation, and low survival rates are reported for such vaguely defined cases (e.g., [7,22,28,33,35]—but see [13] for a detailed analysis of admission causes from a veterinary viewpoint). We therefore analysed the survival chances of birds whose physical trauma or injuries affected different body regions and/or organ systems, regardless of the primary causes of the injuries. As seen from the results summarised in Figure 1, damage to the respiratory system, which was attributable to window collisions in three-quarters of the few identified cases, leaves little chance of survival, so euthanasia would probably reduce costs and effort for the WRCs and suffering for the animals. The chances of survival for birds with injuries (mostly fractures) to their legs, wings, or head are better, but still well below average (compare data in [27] for prospects of severe and very severe cases). Head injuries appear to be typical for collision victims, as three-quarters of the 151 documented cases involve birds that collided with obstacles (mostly windows). Such accidents also lead to a relatively high percentage of injuries to legs and wings (8% and 15% of cases, respectively), but the primary causes of these two injuries were largely unknown in our material. In contrast, birds taken into care due to feather damage were released in 68% of the 34 cases, although they remained in care for a relatively long time, averaging 16 days.

### 4.3. Differences in Rehabilitation Duration of Released Birds

The rehabilitation of wild animals in WRCs is a costly, time-consuming, and sometimes emotionally draining process for the staff involved. Accordingly, some studies ([9,27] with further references) aimed to evaluate the cost–benefit ratio of care measures from the perspective of WRC management, although formalised and generalised indicators, e.g., for triage decisions, are problematic in our opinion for ethical and practical reasons. However, while from the perspective of animal and species protection, survival rates of birds in care can be considered the main criterion for the usefulness of care expenses, the duration of the necessary hospitalisation may be more important from the perspective of a cost–benefit analysis for a WRC. Thus, the time (days) of the rehabilitation stay in a WRC might be used as the basic estimator for assessing the cost of the rehabilitation process, while, for instance, [9] the calculated species/bird group-specific cumulative duration of rehabilitation days can be used as an indicator for rehabilitation effort, [27] expressed a cost–benefit index as the number of released animals per Euro and day. In this study, we only examined the influence of the reason for admission on the length of care (in days) for individual birds that could be released, as no meaningful comparisons between the reasons for admission or bird groups were possible for deceased birds due to the uniform median values (see Table 1).

However, the effect of the admission cause on the necessary duration of care was obvious in our sample as well, cf. [9] with further references. Regardless of the reasons for admission and across all bird groups, patients who were eventually discharged required an average of 15 days (median 11 days) of treatment, but more than a quarter (29%) of individuals remained in care for only 3 days or less, and almost half (49%) for a maximum of 10 days. These are relatively low figures compared to some other comparable WRC studies which report medians of 48 [7], 36 [9], 28–30 [2], 24 [1], and 17 [27] days, for example. Comparatively short treatment stays become even more apparent when comparing stays based on specific reasons for admission and/or individual bird groups/species with the available literature data.

For example, the recovery of victims of building collisions at the Alpenzoo took an average of 10 days (mean; median 3 days), with 70% of birds being released within 10 days, compared to an average of 13 days of treatment (median: 7 days) and only 51% of patients being released within the first 10 days of treatment in a New York study [2]. For other reasons for admission, see, e.g., [9]. The average length of care for released orphaned swifts and passerines was, for example, 19 and 26 days, respectively, at a Catalan WRC [27], compared to 16 days for both bird groups at the Alpenzoo.

We attribute the success of bird care, which is evident in the comparatively short length of stay at the Innsbruck Alpenzoo, to the very elaborate and varied diet and species-appropriate accommodation of the birds in care. These special features are mainly attributable to the legacy of the late Ellen Thaler, whose expertise in bird care is renowned among experts [36,37]. Unfortunately, none of the studies cited provide detailed information on the type of accommodation and feeding of the birds in care, even though these aspects are central to success. We therefore suggest that more attention be paid to these methodological aspects in the future and forthcoming studies.

### 4.4. Differences in the Outcomes of Hospitalisation Between Bird Groups

In line with many other studies [1,6,7,9,17,27,28,33], we found large differences in survival rates between different systematic bird groups and/or ecological guilds, as well as differences within certain groups depending on the cause of admission. However, it is difficult to compare rehabilitation success between different studies because the classification of birds into groups differs in part from our approach or because group-specific survival rates are not always reported. In our sample, rehabilitation success was highest for swifts and swallows, followed by waterfowl (Anseriformes), and it was also comparatively high for raptors and corvids.

We believe that this is partly due to the prevalence of similar reasons for admission (orphanage, ‘weakness’, and ‘undetermined’) for the dominant species in these groups. These reasons for admission usually relate to individuals in good initial condition and/or with only minor injuries. Such cases or birds not only generally have higher release rates (in this study, compare, e.g., [7,9,27]), but in our case, also benefit from the specific experience of the bird keepers and the resources (variety of food, accommodation) of the zoo. In our study, the group of hospitalised aerial insectivores is dominated by Common Swifts and, to a lesser extent, House Martins *Delichon urbicum* and Barn Swallows *Hirundo rustica*, most of which are admitted either as nestlings and/or in an emaciated, exhausted state. Anseriformes are dominated in our sample by Mallards *Anas platyrhynchos* and Goosanders *Mergus merganser*, and a high proportion of these species are also recorded as young, dependent ducklings (at least 55%). The situation is similar for raptors and corvids, which are dominated by only three species (71% of recordings) or even just one species (Carrion crow). Within these groups, species with survival rates above the group average, such as the Kestrel and the Carrion crow, are represented in our sample with a relatively high proportion of nestlings or fledglings.

Surprisingly, however, small passerines, which are also frequently brought in as nestlings (41%), and have a high proportion of unidentified cases (24%) in the records, which probably also include many nestlings or weakened birds, have relatively low survival rates (45%) at the Alpenzoo (but see [6,27] with 61% and 75% release rates for passerines). These low figures can be explained in part by differences between the survival rates of adult birds (36%) and nestlings (54%), which also differ between the two groups depending on their feeding habits (Figure 3). Even the care of young granivorous/herbivorous songbirds led to below-average results (44% survival rate), underscoring the relative difficulties in rearing young birds of this food group, which have long been known to experienced bird keepers. Furthermore, small songbirds are also the most frequent victims of cat attacks, both in absolute and proportional terms, and generally seem to have the lowest chances of survival (see above).

Also, the chances of survival for birds injured by collisions or otherwise traumatised small birds, including both Nonpasserines (including woodpeckers) and small songbirds, were lower on average than those of all other groups. Woodpeckers, especially, had very low chances of survival, which differed significantly from those of most other groups. It should be noted that victims of building collisions/window strikes, which generally have a rather low chance of survival anyway (this study, see also, e.g., [2,4,6,7,9,13,17,27,33,34]), occur disproportionately frequently (29% of all cases) and are the main cause of admission for Woodpeckers. Only in medium-sized Nonpasserines was this proportion even higher (37%), but here the Woodcock *Scolopax rusticola* dominates the admittance protocols, accounting for 52% of all cases in this group, with 71% of its individuals admitted as victims of collisions.

## 5. Conclusions

Urbanisation and suburbanisation are advancing worldwide, constantly increasing the risk of wild animals being injured in unfamiliar structures or affected by human activities. Accordingly, the number of birds and the diversity of bird species that need to be admitted to WRCs are also increasing worldwide from year to year. This process can even be observed in our study area that is located in the centre of the Alps, which is an area that is generally considered a biodiversity hotspot and of great value for nature conservation. However, in the increasingly urbanised Inn Valley, most wild bird records are directly or indirectly related to human influences, affecting all bird groups and species. We believe that our data, which cover 137 species, and, to our knowledge, have been collected over a longer period than in any other WRC, are particularly valuable for assessing long-term trends in the problems faced by wild birds in urbanised areas. In addition, they demonstrate the need for and possibilities of professionally run WRCs in such landscapes. We conclude that zoos, that do not normally function as WRCs due to understandable reasons of cost and labour, can play an important role in regional conservation and in promoting regional awareness of wild birds if they participate in rescue efforts. At the least, they should share their specific expertise in bird care and their knowledge of species-appropriate feeding and housing of wild birds with local WRCs. We also believe it is important that future reports on the successes and failures of hospitalisations focus more strongly on these aspects.

## Figures and Tables

**Table 1:** Total number of admitted birds and bird species at Innsbruck Alpenzoo (1988–2020) and overview of the rehabilitation outcomes according to different admission causes. Values represent the percentage and numbers of birds which could be released (= survival rate) based on the full data set of admissions with the known outcome of hospitalisation (*n* = 4542). The duration of rehabilitation until release and the care days in birds that deceased or had to be euthanised during their care are given in columns 5 and 6, respectively. The total numbers of birds per cause in columns 5 and 6 are only a subset of the total numbers in column 2 because the durations of care were not exactly registered in 1111 cases. Rehabilitation/care durations are presented in days as median with MAD (median absolute deviation) and (min–max) values. Hospitalisation durations of birds which could be released or died after basic care during the day of admittance were counted as 0.5 days. Significance levels of differences between single causes compare with Table S1. # = hospitalisation durations of birds which could be released or died after basic care during the day of admittance were counted as 0.5 days.

Categories/Causes for Admission	Number of Admitted Birds	Number of Admitted Species	Survival Rate % (Individuals)	Rehabilitation Duration Days ^#^/*n* Released Birds	Care Duration Days ^#^/*n* Dead Birds
Admitted wild birds from the study area *^1^	5301	145	-	-	-
–outcome of hospitalisation registered–total	4542	137	51.0 (2313)	11.0 ± 9.0 (0.5–183)/1633	1.0 ± 0.5 (0.5–125)/1807
–orphaned or stranded nestlings	1443	62	63.4 (915)	15.5 ± 8.5 (0.5–90)/644	1.0 ± 0.5 (0.5–60)/443
–cause of admission “undetermined” *^2^	1272	111	55.3 (703)	7.0 ± 6.0 (0.5–153)/487	1.0 ± 0.5 (0.5–125)/469
–collisions with windows/building *^3^	582	88	41.9 (244)	3.0 ± 2.5 (0.5–117)/193	1.0 ± 0.5 (0.5–89)/276
–physical trauma (unknown causes) *^4^	558	81	35.5 (199)	13.0 ± 9.0 (0.5–183)/156	1.0 ± 0.5 (0.5–111)/284
–“weak” birds *^5^	294	58	43.9 (129)	10.0 ± 7.0 (0.5–89)/66	1.0 ± 0.5 (0.5–34)/130
–cat-. pet and raptor attacks *^6^	281	60	27.1 (76)	12.0 ± 9.0 (0.5–54)/63	1.0 ± 0.5 (0.5–36)/168
–vehicle collision *^7^	63	20	39.7 (25)	7.0 ± 7.0 (0.5–100)/14	1.0 ± 0.5 (0.5–15)/23
–anthropogenic structures *^8^	27	10	63.0 (17)	3.0 ± 2.5 (0.5–31)/9	0.8 ± 0.3 (0.5–21)/6
–persecution (gunshots, poisoning, traps)	22	13	36.4 (8)	6.0 ± 0 (6)/1	1.0 ± 0.5 (0.5–4)/10

*^1^ including data sets of 44 small passerine nestling birds of questionable species affiliation, excluding feral pigeons and allochthonous captive escapes. *^2^ reasons for rescue and type of potential trauma unknown or not registered. *^3^ mostly victims of window strikes (578 cases, 88 species). *^4^ cases with obvious or diagnosed injuries/traumata (mostly fractures of wings, legs, or beaks). Primary causes of injury unclear. *^5^ poor overall condition: emaciated, exhausted birds able to fly including fledglings (reasons for weakness partly unknown—no evident injuries or causes for weakness). *^6^ mainly cat attacks (252 cases 53 species); in addition, 17 attacks by ravens (*Corvus corax*) or Carrion crows (*Corvus corone*), eight attacks by unknown birds of prey, and three dog attacks. *^7^ mostly car accidents; including three collisions with trains. *^8^ entanglement in any man-made structures, i.e., fences, barbed wires, power lines, nets, cellar shafts, including four oiled birds.

**Table 2:** Differences in survival rates of birds admitted to Innsbruck Alpenzoo between 1988 and 2020 due to various main causes. Data from three periods (decades) of 12, 11, and 10 years are compared. Significant differences in the proportion of surviving birds are marked: * = *p* < 0.05; ** = *p* < 0.01; ns = not significant; Chi^2^ tests. Collisions = collisions with buildings (mostly window strikes); attacks = victims of attacks by domestic animals or raptors (mostly cats). Cases with small sample sizes (see Table 1) were not analysed separately but were summarised under “all causes”.

→ Periods	1988–1999		↔	2000–2010		↔	2011–2020		↔	1988–1999
Admission Causes ↓	Hospitalised (All Cases)	Survived *n* (%)		Hospitalised (All Cases)	Survived *n* (%)		Hospitalised (All Cases)	Survived: *n* (%)		Survived %
nestlings	241	168 (69.7)	*	426	259 (60.8)	ns	776	488 (62.9)	ns	69.7
“weak” birds	106	60 (56.6)	*	81	31 (38.3)	ns	107	38 (35.5)	**	56.6
phys. trauma	114	48 (42.1)	ns	156	51 (32.7)	ns	286	98 (34.3)	ns	42.1
collisions	189	96 (50.8)	ns	119	49 (41.2)	ns	272	100 (36.8)	**	50.8
attacks	34	11 (32.4)	ns	47	12 (25.5)	ns	196	53 (27.0)	ns	32.4
all causes	1063	581 (54.7)	*	1260	631 (50.1)	ns	2205	1101 (49.9)	*	54.7

**Table 3:** Species- and group-specific numbers of admission causes and hospitalisation outcomes for wild birds (*n* = 4542) admitted to Innsbruck Alpenzoo between 1988 and 2020. Only species with a minimum of 70 hospitalised individuals (*n* = 15) are shown separately. Highlighted rows (grey) indicate numbers for higher-level systematic bird groups or ecological guilds, including the respective species separately listed as well as other species not shown in the table. Numbers of released individuals are given in brackets for each admission cause and species or group. Total numbers and percentages of birds which survived the hospitalisation are presented per bird species/group in the last column. For overall survival rates per admission cause, compare with Table 1.

Species/Group (*n* Species)	Orphaned	Unknown	Collision	Trauma	Weak	Attacked	Vehicle	Structure	Persecuted	Total	Survived (%)
Common Blackbird	239 (159)	130 (72)	42 (19)	54 (22)	8 (2)	104 (24)	2 (0)	0 (0)	0 (0)	579	290 (50.1)
House Sparrow	195 (87)	91 (43)	27 (6)	16 (6)	21 (1)	31 (6)	0 (0)	0 (0)	0 (0)	381	149 (39.1)
Great Tit	69 (45)	19 (10)	7 (3)	8 (1)	4 (0)	14 (6)	1 (1)	0 (0)	0 (0)	122	66 (54.1)
Greenfinch	33 (13)	21 (4)	9 (3)	10 (1)	7 (1)	6 (4)	0 (0)	0 (0)	0 (0)	86	26 (30.2)
Chaffinch	21 (11)	24 (19)		14 (12)	2 (0)	19 (9)	1 (0)	0 (0)	0 (0)	83	42 (50.6)
Blackcap	39 (8)	18 (8)	7 (3)	3 (2)	2 (0)	7 (1)	0 (0)	0 (0)	0 (0)	76	22 (28.9)
Small Passerines (57) *	864 (466)	496 (244)	256 (96)	175 (71)	65 (10)	222 (56)	7 (3)	3 (1)	O (0)	2087	946 (45.3)
Carrion Crow	132 (105)	97 (53)	23 (10)	95 (25)	25 (11)	7 (4)	4 (1)	1 (1)	5 (0)	388	209 (53.9)
Corvids (7)	157 (122)	121 (61)	35 (12)	120 (33)	32 (13)	13 (5)	4 (1)	1 (1)	6 (0)	488	247 (50.6)
Common Swift	176 (149)	161 (117)	11 (3)	40 (7)	79 (55)	9 (5)	0 (0)	5 (2)	0 (0)	481	338 (70.3)
House Martin	32 (20)	36 (27)	1 (0)	3 (1)	5 (0)	1 (1)	0 (0)	0 (0)	0 (0)	78	48 (61.5)
Barn Swallow	14 (12)	35 (17)	5 (0)	5 (0)	9 (1)	2 (0)	0 (0)	1 (1)	0 (0)	71	31 (43.7)
Swifts and Swallows (5)	226 (184)	239 (164)	18 (4)	49 (9)	95 (56)	12 (5)	0 (0)	6 (3)	0 (0)	645	425 (65.9)
Great Spotted Woodpecker	20 (16)	21 (8)	23 (6)	9 (1)	5 (1)	8 (1)	0 (0)	0 (0)	0 (0)	86	33 (38.4)
Woodpeckers (5)	27 (17)	28 (9)	35 (14)	16 (4)	5 (1)	9 (1)	2 (1)	1 (0)	0 (0)	122	47 (38.5)
Common Kestrel	61 (51)	41 (31)	24 (11)	40 (25)	11 (6)	2 (1)	5 (1)	11 (10)	1 (0)	195	136 (69.7)
Common Buzzard	2 (1)	55 (29)	37 (15)	23 (10)	16 (7)	3 (2)	34 (15)	2 (0)	3 (1)	174	80 (46.0)
Eurasian Sparrowhawk	1 (0)	63 (30)	55 (27)	27 (6)	6 (2)	1 (1)	4 (2)	0 (0)	0 (0)	157	67 (42.7)
Raptors (19) #	82 (69)	228 (139)	162 (76)	124 (54)	61 (28)	12 (8)	46 (19)	20 (13)	11 (5)	744	411 (55.2)
Mallard	47 (35)	21 (16)	3 (1)	19 (7)	0 (0)	2 (1)	0 (0)	0 (0)	0 (0)	92	60 (65.2)
Anseriformes (6)	77 (49)	29 (21)	5 (3)	25 (11)	1 (1)	2 (1)	1 (0)	1 (1)	1 (1)	141	87 (61.7)
Small Nonpass. (17) +	9 (7)	71 (38)	19 (10)	26 (9)	11 (2)	5 (0)	1 (1)	0 (0)	2 (0)	144	67 (46.5)
Med. Nonpass. (15) ++	0 (0)	42 (15)	47 (31)	17 (8)	15 (12)	6 (0)	1 (0)	0 (0)	0 (0)	128	66 (51.6)
Larger Nonpass. (7) +++	1 (1)	18 (12)	5 (0)	6 (0)	9 (6)	0 (0)	1 (0)	2 (0)	2 (2)	43	21 (48.8)

* = including members of 20 songbird families; # = including 396 Accipitriformes (eight species), 229 Falconiformes (four spp.), 119 Strigiformes (seven spp.); + = all other nonpasserine birds with adult weight below 250 g: small waders, rails, doves, Cuckoo *Cuculus canorus*, Kingfisher *Alcedo atthis*, Bee-eater *Merops apiaster*, Hoopoe *Upupa epops*, Nightjar *Caprimulgus europaeus*, Little Grebe *Tachybabtus ruficollis*; ++ = all other medium-sized Nonpasserines weighing between 250 and 1000 g: Galliformes, Podicipedidae, Rallidae, Scolopacidae, Laridae, Woodpigeon *Columba palumbus*; +++ = other larger Nonpasserines with >1 kg adult weight: Capercaillie *Tetrao urogallus*, Grey Heron *Ardea cinerea*, Bittern *Botaurus stellaris*, White Stork *Ciconia ciconia*, Cormorant *Phalacrocorax carbo*, Black-throated Diver *Gavia artica*, Silver Gull *Larus argentatus*. For detailed species lists and admission numbers, see Table S1 in [8].

## Data Availability

The original contributions presented in this study are included in the article/Supplementary Material. Further inquiries can be directed to the corresponding author.

## Supplementary Materials

The following supporting information can be downloaded at https://www.mdpi.com/article/10.3390/ani16020221/s1; Table S1: Significance levels of differences in survival rates between main groups of admission causes of birds admitted at Innsbruck Alpenzoo, 1988–2020; Table S2: Significance levels of differences in the number of days under care between main groups of admission causes for successfully hospitalised and released birds admitted at Innsbruck Alpenzoo, 1988–2020; Table S3: Significance levels of differences in survival rates between main bird groups admitted at Innsbruck Alpenzoo, 1988–2020.
