# Supplementary material for: Survival Prospects of Wild Birds Depending on the Type of Injury and Other Stressors Leading to Hospitalisation: A Long-Term (1988–2020) Retrospective Study from an Urbanised Area of the Alps
Source: Animals (Basel). 2026 Jan 12;16(2):221. doi: 10.3390/ani16020221 (PMC12838066; doi:10.3390/ani16020221)
Supplement: Supplementary file 1 [file animals-16-00221-s001.zip › animals-4051067-supplementary.pdf]

Survival Prospects of Wild Birds Depending on the Type of Injury and Other Stressors Leading to Hospitalisation: A Long-Term (1988–2020) Retrospective Study from an Urbanised Area of the Alps

Christiane Böhm, Molinia Wilberger, Armin Landmann*

Supplementary material Tables S1–S3

Table S1: Significance levels of differences in survival rates between main groups of admission causes of birds admitted at Innsbruck Alpenzoo 1988–2020. Causes are ranked in descending order of survival percentages:1 = orphaned nestlings; 2 = anthropogenic structures; 3 = cause of admission undetermined; 4 = weakness; 5 = window/building collisions; 6 = vehicle collisions; 7 = persecution; 8 = physical trauma; 9 = cat-, pet- & raptor attacks (see Table 1 for details). One way ANOVA, pairwise multiple comparison procedures (Holm-Sidak method). Significance levels of chi^2-tests with Yates correction: are given in brackets if they differ from the ANOVA results. ns = not significant, p< 0,05; * = p< 0,05; ** = p< 0,01; *** = p< 0,001.

Cause	1	2	3	4	5	6	7	8	9
1	–	ns	***	***	***	**	ns (*)	***	***
2		–	ns	ns	ns	ns	ns	ns (*)	**
3			–	**	***	ns (*)	ns	***	***
4				–	**	ns	ns	ns (*)	***
5					–	ns	ns	ns (*)	***
6						–	ns	ns	ns
7							–	ns	ns
8								–	ns
9									–

Table S2: Significance levels of differences in the number of days under care between main groups of admission causes for successfully hospitalised and released birds admitted at Innsbruck Alpenzoo 1988–2020 (see Table 1). Causes are ranked in descending order of the median of care days (see Table 1): 1 = orphaned nestlings; 2 = physical trauma; 3 = cat-, pet- & raptor attacks; 4 = "weakness"; 5 = cause of admission undetermined; 6 = vehicle collisions; 7 = window/building collisions; 8 = anthropogenic structures; persecution not calculated (only 1 case). Results of pairwise Mann-Whitney Rank Sum Tests. ns = not significant; * = p< 0,05; ** = p< 0,01; *** = p<0,001. Also tested with Kruskal-Wallis One Way Analysis of Variance on Ranks (p <0,05).

Cause	1	2	3	4	5	6	7	8
1	–	ns	ns	***	***	**	***	*
2		–	ns	*	*	*	***	*
3			–	ns	*	ns	***	ns
4				–	ns	ns	ns	ns
5					–	ns	*	ns
6						–	ns	ns
7							–	ns
8								–

Table S3: Significance levels of differences in survival rates between main bird groups admitted at Innsbruck Alpenzoo 1988–2020 irrespective of admission causes. Bird groups are ranked in descending order of survival percentages:1 = Swifts & Swallows; 2 = Anseriformes; 3 = Raptors; 4 = Corvids; 5 = other medium sized Nonpasserines; 6 = other large sized Nonpasserines; 7 = other small sized Nonpasserines; 8 = small Passerines; 9 = Woodpeckers (see Table 3 for details). One way ANOVA, pairwise multiple comparison procedures (Holm-Sidak method). Significance levels of chi^2-tests with Yates correction: are given in brackets if they differ from the ANOVA results. ns = not significant; * = p< 0,05; ** = p< 0,01; *** = p< 0,001. Detailed species lists and admission numbers see Table S1 in [8].

Group	1	2	3	4	5	6	7	8	9
1	–	ns	**	***	ns (*)	ns (*)	***	***	***
2		–	ns	ns	ns	ns	ns (*)	**	**
3			–	ns	ns	ns	ns	***	*
4				–	ns	ns	ns	ns	ns (*)
5					–	ns	ns	ns (*)	***
6						–	ns	ns	ns
7							–	ns	ns
8								–	ns
9									–
